# Tuberculosis in Hospitalized Patients With Human Immunodeficiency Virus: Clinical Characteristics, Mortality, and Implications From the Rapid Urine-based Screening for Tuberculosis to Reduce AIDS Related Mortality in Hospitalized Patients in Africa

**DOI:** 10.1093/cid/ciz1133

**Published:** 2019-11-29

**Authors:** Ankur Gupta-Wright, Katherine Fielding, Douglas Wilson, Joep J van Oosterhout, Daniel Grint, Henry C Mwandumba, Melanie Alufandika-Moyo, Jurgens A Peters, Lingstone Chiume, Stephen D Lawn, Elizabeth L Corbett

**Affiliations:** 1 Tuberculosis Centre, London School of Hygiene and Tropical Medicine, London, United Kingdom; 2 Clinical Research Department, London School of Hygiene and Tropical Medicine, London, United Kingdom; 3 Malawi-Liverpool-Wellcome Trust Clinical Research Programme, University of Malawi College of Medicine, Blantyre, Malawi; 4 Department of Infectious Disease Epidemiology, London School of Hygiene and Tropical Medicine, London, United Kingdom; 5 University of the Witwatersrand, Johannesburg, South Africa; 6 Department of Medicine, Edendale Hospital, University of KwaZulu-Natal, Pietermaritzburg, South Africa; 7 Dignitas International, Zomba, Malawi; 8 Department of Medicine, College of Medicine, University of Malawi, Blantyre, Malawi; 9 Department of Clinical Sciences, Liverpool School of Tropical Medicine, Liverpool, United Kingdom

**Keywords:** HIV, TB, hospital inpatients, mortality, urine LAM

## Abstract

**Background:**

Tuberculosis (TB) is the major killer of people living with human immunodeficiency virus (HIV) globally, with suboptimal diagnostics and management contributing to high case-fatality rates.

**Methods:**

A prospective cohort of patients with confirmed TB (Xpert MTB/RIF and/or Determine TB-LAM Ag positive) identified through screening HIV-positive inpatients with sputum and urine diagnostics in Malawi and South Africa (Rapid urine-based Screening for Tuberculosis to reduce AIDS Related Mortality in hospitalized Patients in Africa [STAMP] trial). Urine was tested prospectively (intervention) or retrospectively (standard of care arm). We defined baseline clinical phenotypes using hierarchical cluster analysis, and also used Cox regression analysis to identify associations with early mortality (≤56 days).

**Results:**

Of 322 patients with TB confirmed between October 2015 and September 2018, 78.0% had ≥1 positive urine test. Antiretroviral therapy (ART) coverage was 80.2% among those not newly diagnosed, but with median CD4 count 75 cells/µL and high HIV viral loads. Early mortality was 30.7% (99/322), despite near-universal prompt TB treatment. Older age, male sex, ART before admission, poor nutritional status, lower hemoglobin, and positive urine tests (TB-LAM and/or Xpert MTB/RIF) were associated with increased mortality in multivariate analyses. Cluster analysis (on baseline variables) defined 4 patient subgroups with early mortality ranging from 9.8% to 52.5%. Although unadjusted mortality was 9.3% lower in South Africa than Malawi, in adjusted models mortality was similar in both countries (hazard ratio, 0.9; *P* = .729).

**Conclusions:**

Mortality following prompt inpatient diagnosis of HIV-associated TB remained unacceptably high, even in South Africa. Intensified management strategies are urgently needed, for which prognostic indicators could potentially guide both development and subsequent use.

## Teaser

Among 322 HIV-positive inpatients with tuberculosis, 2-month mortality was 31%, and dissemination was common. Older age, being male, taking antiretroviral therapy at admission, poor nutritional status, and positive urine diagnostics were associated with mortality. Interventions to reduce mortality are needed.

**(See the Editorial Commentary by Walsh and Koenig on pages 2627–9.)**

Human immunodeficiency virus (HIV)–associated tuberculosis (TB) disease is a leading cause of mortality, accounting for 370 000 deaths globally in 2016 [1]. Much of this burden resides in patients admitted to hospitals in settings of high HIV prevalence in Africa [2]. A meta-analysis estimated that TB caused 24% of admissions and 27% of deaths among HIV-positive inpatients, with 30% case fatality [3]. Postmortem data suggest an even greater burden, as almost half of fatal TB remains undiagnosed [4]. However, these data predate widespread access to antiretroviral therapy (ART) and improved diagnostics such as the Xpert MTB/RIF assay.

There remains a scarcity of data on factors associated with mortality in HIV/TB. Postmortems suggest TB as the predominant cause of death [4], but substantial comorbidity from other opportunistic infections and noninfectious conditions means that death *with* confirmed TB does not necessarily imply death *from* TB. Observational cohorts report low CD4 cell count and older age as associated with mortality, but these data mainly relate to ART-naive individuals, and include patients without bacteriologically confirmed TB disease [5–7]. Disseminated HIV/TB, as indicated by *Mycobacterium tuberculosis* detection in blood or urine, is also common and is an independent predictor of mortality [8–10].

Understanding factors associated with mortality in hospitalized patients with HIV/TB in the context of high ART coverage, rapid TB diagnostics with better yield, and prompt TB treatment could help inform strategies to identify high-risk patients, and interventions to reduce mortality. Here we describe characteristics of patients diagnosed with TB from the Rapid urine-based Screening for Tuberculosis to reduce AIDS Related Mortality in hospitalized Patients in Africa (STAMP) randomized controlled trial of urine-based TB screening in HIV-positive inpatients in Malawi and South Africa [11].

Our specific aims were to describe the clinical phenotypes, mortality, and risk factors for mortality in hospitalized HIV/TB patients; the prevalence and mortality of disseminated TB; and the impact of ART and study site on mortality.

## METHODS

This prospective cohort study was nested within the STAMP trial [11, 12], which recruited adult HIV-positive patients, irrespective of clinical presentation, at admission to medical wards in 2 hospitals in Malawi and South Africa. Upon enrollment, patients were randomized to TB screening using sputum testing alone (standard of care [SOC]), or sputum and urine testing (intervention). Primary outcome was all-cause mortality at 2 months, and secondary outcomes included TB diagnosis and treatment. Patients were excluded if they had TB treatment within 12 months, isoniazid preventative therapy within 6 months, were unable to provide informed consent, or been admitted for >48 hours at the time of screening.

Trial procedures are outlined in the Supplementary Methods [11, 12]. After enrollment, urine and spontaneously expectorated sputum samples were obtained and tested for TB according to trial arm. Patients allocated to the intervention arm underwent sputum testing (if produced) with the Xpert MTB/RIF assay (Xpert), unconcentrated urine was tested with the Determine TB-LAM Ag assay (TB-LAM), and concentrated urine was tested with Xpert [13]. Patients allocated to the SOC arm had sputum Xpert testing only: with urine immediately frozen. Clinical events during admission were recorded, and patients were followed up once at 2 months by interview. Patients not attending follow-up were contacted by telephone and/or home visit, with interview of next of kin to establish vital status if necessary.

Patients diagnosed with TB in the SOC trial arm (ie, no real-time urine TB testing) had TB-LAM and Xpert testing performed on stored urine (Supplementary Methods). Patients were enrolled in this substudy if they were diagnosed with TB and had positive laboratory tests (≥1 positive specimen on microscopy for acid-fast bacilli, Xpert, culture, or TB-LAM) on any sample. Patients diagnosed or treated for TB without positive tests were excluded. The study was approved by the research ethics committee of the London School of Hygiene and Tropical Medicine, and local research ethics committees in Malawi and South Africa.

### Definitions

Patients were defined as “clinically suspected TB” if TB was recorded in the admitting differential diagnosis. Disseminated urinary TB was defined by any positive urine TB assay (TB-LAM or Xpert), and nondisseminated TB was a positive sputum TB assay with negative urine TB assays.

A urine “TB score” was calculated based on the number of positive urine TB tests (TB-LAM or Xpert, possible values 0–2) [14]. The Supplementary Methods outline other definitions.

### Statistical Analysis

Mortality risk was calculated 56 days from admission. All baseline demographics, clinical variables, laboratory results, and physiological measurements were considered for association with mortality using Cox proportional hazards models. An explanatory model was built excluding the most proximal factors on the causal pathway to mortality (notably, functional impairment). Separate models were built to assess associations of recruitment site with mortality. Models used stepwise backward elimination (variables with *P* > .1 were excluded) and were restricted to complete cases. Nonlinearity of continuous variables was assessed using fractional polynomials.

Clinical phenotypes of HIV/TB patients were identified using unsupervised (ie, without mortality outcome) hierarchical cluster analysis, with the number of clusters determined by stopping rules. Clusters were then described by comparing means and proportions to the overall population, and associations with mortality assessed using Kaplan-Meier curves, and Cox regression. The Supplementary Methods describe the analysis in further detail.

## RESULTS

### Patient Characteristics

Between October 2015 and September 2017, 506 HIV-positive patients were diagnosed with TB, of whom 322 were laboratory-confirmed and included in this analysis. Similar numbers were from Malawi (155 [48.1%]) and South Africa (167 [51.9%]), with two-thirds (63.7%) clinically suspected to have TB (Table 1).

**Table 1.** Baseline Characteristics in Patients With Laboratory-Confirmed Human Immunodeficiency Virus/Tuberculosis (TB), Overall and Stratified by Disseminated Urinary TB

				Disseminated TB		
Characteristic	Variable	All HIV/TB (N = 322)	Yes (n = 251)	No (n = 71)	*P* Value^a^
Age	Mean (SD)	37.2	(10.5)	36.8	(10.3)	38.7	(11.3)	.175
Sex	Male	175	(54.3)	131	(52.2)	44	(62.0)	.144
	Female	147	(45.7)	120	(47.8)	27	(38.0)	
Site	Malawi	155	(48.1)	131	(52.2)	24	(33.8)	.006
	South Africa	167	(51.9)	120	(47.8)	47	(66.2)	
Smoking status	Current	35	(10.9)	26	(10.4)	9	(12.7)	.009
	Former	77	(23.9)	51	(20.3)	26	(36.6)	
Comorbidity	Yes	65	(20.2)	49	(19.5)	16	(22.5)	.577
New diagnosis of HIV	Yes	55	(17.1)	43	(17.1)	12	(16.9)	.964
ART status^b^	Naive	34	(12.7)	25	(12.0)	9	(15.3)	
	Current	214	(80.1)	165	(79.3)	49	(83.1)	.167
	Interrupted	19	(7.1)	18	(8.7)	1	(1.7)	
Time on ART, y^c^	Median (IQR)	1.5	(5.0)	0.9	(4.2)	1.6	(5.2)	.962
Second-line ART ^c^	Yes	6	(2.8)	2	(2.5)	4	(4.1)	.547
Cough	Yes	234	(72.7)	175	(69.7)	59	(83.1)	.026
Fever	Yes	228	(70.8)	182	(72.5)	46	(64.8)	.206
Night sweats	Yes	170	(52.8)	136	(54.2)	34	(47.9)	.348
Weight loss	Yes	293	(91.0)	227	(90.4)	66	(93.0)	.513
≥1 WHO TB symptom	Positive	317	(98.4)	247	(98.4)	70	(98.6)	.911
Duration of illness, d	Median (IQR)	14	(21)	14	(21)	14	(21)	.873
Previous TB treatment	Yes	73	(22.7)	53	(21.1)	20	(28.2)	.21
EQ5D mobility	Some problems	163	(50.6)	129	(51.4)	34	(47.9)	.067
	Confined to bed	77	(23.9)	65	(25.9)	12	(16.9)	
EQ5D self-care	Some problems	129	(40.1)	107	(42.6)	22	(31.0)	< .001
	Unable to wash/ dress	86	(26.7)	75	(29.9)	11	(15.5)	
EQ5D usual activities	Some problems	91	(28.3)	72	(28.7)	19	(26.8)	.002
	Unable to perform	165	(51.2)	138	(55.0)	27	(38.0)	
EQ5D health score	Mean (SD)	52.1	(13.6)	48.0	(14.2)	54.0	(11.4)	< .001
BMI, kg/m^2^	Median (IQR)	18.2	(5.0)	18.0	(4.6)	19.4	(6.2)	.049
MUAC, cm	Median (IQR)	20	(5.5)	20	(4.5)	21	(6.5)	.039
Karnofsky score	Median (IQR)	50	(10)	50	(20)	60	(20)	.001
	≤40	74	(23.0)	65	(25.9)	9	(12.7)	.019
Blood pressure, mm Hg	Median SBP	102	…	101	…	106	…	.009
	Median DBP	67	…	66	…	70	…	.060
Heart rate, beats/min	Mean (SD)	104.7	(20.8)	105.5	(21.1)	101.8	(19.5)	.189
Respiratory rate, breaths/min	Mean (SD)	23.3	…	22.8	…	23.4	…	.265
Oxygen saturation, %	Median (IQR)	96	(4)	96	(4)	96	(4)	.991
WHO danger sign	Yes	139	(43.2)	117	(46.6)	22	(31.0)	.019
Sepsis	Yes	77	(23.9)	69	(27.5)	8	(11.3)	.005
Hemoglobin, g/dL	Mean (SD)	8.8	(27.7)	8.4	(26.1)	10.2	(28.9)	< .001
Anemia	Severe	133	(41.3)	115	(45.8)	18	(25.4)	.001
	Moderate	117	(36.5)	91	(77.8)	26	(22.2)	
	Mild	42	(13.1)	27	(64.3)	15	(35.7)	
CD4 count, cells/μL	Median (IQR)	74.5	(182)	61	(157)	129	(295)	.002
	<100	188	(58.4)	155	(61.8)	33	(46.5)	.017
CRP	Median (IQR)	138	(112)	141.5	(106)	117	(136)	.619
WHO stage	1 or 2	35	(10.9)	28	(11.2)	7	(9.9)	
	3	111	(34.5)	78	(31.1)	33	(46.5)	.052
	4	176	(54.7)	145	(57.8)	31	(43.7)	
Clinically suspected TB	Yes	205	(63.7)	147	(58.6)	58	(81.7)	< .001

Data are presented as no. (%) unless otherwise stated.

Abbreviations: ART, antiretroviral therapy; BMI, body mass index; CRP, C-reactive protein; DBP, diastolic blood pressure; EQ5D, EurQol 5 dimension; HIV, human immunodeficiency virus; IQR, interquartile range; MUAC, mid-upper arm circumference; SBP, systolic blood pressure; SD, standard deviation; TB, tuberculosis; WHO, World Health Organization.

^a^Comparing disseminated TB yes/no, calculated using χ ^2^ test for proportions, *t* test for means, and Wilcoxon rank-sum test for medians.

^b^Restricted to patients with known HIV diagnosis.

^c^Restricted to patients reporting current ART use. Missing data: 1 missing hemoglobin, 2 missing second-line ART regimen, 93 missing CRP.

At admission, median CD4 cell count was 75 cells/µL, and 93% had advanced HIV (as defined by World Health Organization [WHO]). One hundred thirty-nine (43.2%) patients had ≥1 WHO danger sign, and 77 (23.9%) had signs of sepsis. Patients had substantial functional impairment, with 165 (51.2%) being unable to perform usual activities and 74 (23.0%) assessed as severely disabled (Karnofsky score ≤40), and poor nutritional status (median body mass index [BMI], 18.2 kg/m^2^). Anemia was very common; only 30 (9.3%) patients had normal hemoglobin levels, and 62 (19.3%) had life-threatening anemia (hemoglobin <6.5 g/dL).

Two hundred sixty-seven (82.9%) patients knew their HIV status before admission, of whom 214 (80.1%) were taking ART. Median duration of ART was 1.0 year (interquartile range [IQR], 0.3–4.2 years) and 68 (21.1%) were on ART for <3 months. Most (58.4% [125/214]) patients reporting current usage had been taking ART for ≥6 months, although their median CD4 cell count was only 96 cells/µL (IQR, 32–319 cells/μL). HIV viral load results were available for 97 of 125 patients on ART for ≥6 months; 48 (49.5%) had >1000 copies/mL (median, 557 000 copies/mL), highly suggestive of ART failure.

### TB Characteristics

TB diagnostic results are outlined in Table 2. Most (242/322 [75.2%]) patients could provide sputum for Xpert testing. Disseminated urinary TB was common (78.0% [251/322]). When restricted to patients in the trial intervention arm, 181 of 212 (85.4%) patients had disseminated TB. One-third (86/251 [34.2%]) of disseminated urinary TB patients were positive on both urine TB-LAM and Xpert assays.

**Table 2.** Tuberculosis Investigations and Results (N = 322)

TB Investigation	No. (%)
Sputum	
Sample sent for TB testing	242 (75.2)
Xpert positive	168 (52.2)
Urine	
Sample sent for TB testing	321 (99.7)
TB-LAM positive	209 (66.1)
Xpert positive	128 (40.5)
Any urine TB test positive	251 (78.0)
Urine TB score	
0	71 (22.0)
1	165 (51.2)
2	86 (26.7)
Chest radiography	
Underwent chest radiography	197 (61.2)
Clinicians report as “consistent with TB”	107 (33.2)
Cerebrospinal fluid^a^	
Tested	45 (14.0)
Consistent with TB	4 (1.2)
Rifampicin-resistant TB	
Xpert rifampicin result available (sputum or urine)	223 (69.3)
Rifampicin resistance detected	11 (4.9)

The percentages are based on all patients (N = 322) as the denominator. Sputum includes study TB screening and routine clinical samples.

Abbreviations: TB, tuberculosis; TB-LAM, Determine TB-LAM Ag assay; Xpert, Xpert MTB/RIF assay.

^a^Cerebrospinal fluid (CSF) testing consistent with TB includes lymphocytes with raised protein or positive Xpert. One patient was missing data for CSF testing. TB score was calculated based on the number of positive urine TB tests (TB-LAM or Xpert, possible values 0–2).

Of 197 patients undergoing chest radiography, 107 (54.3%) were interpreted by clinicians as consistent with TB. Only 4 patients (8.9% of those with cerebrospinal fluid results) were diagnosed with TB meningitis, of whom all had positive urine TB tests (3 Xpert positive, 1 TB-LAM positive).

Patients with disseminated TB were less likely to report a cough or to be clinically suspected of having TB. They were predominantly male and had more functional impairment (bed-bound, unable to perform usual activities, and lower Karnofsky score); worse nutritional status (lower BMI and mid-upper arm circumference [MUAC]); were more likely to have WHO danger signs, sepsis, and severe anemia at presentation; and had lower CD4 cell counts (Table 1).

Eleven patients died before TB treatment initiation, while for those treated, median time from admission to treatment was 2–4 days (IQR, 1 day). Four (1%) patients stopped TB treatment during hospitalization due to treatment side effects. Eleven patients were diagnosed with rifampicin-resistant TB: 6 from sputum Xpert only, 4 from urine Xpert only, and 1 on both urine and sputum Xpert.

### Mortality

Overall, 99 (30.7%) patients died by 56 days. Six patients were lost to follow-up after discharge. Mortality in HIV/TB patients did not differ by STAMP trial arm (*P* = .30), consistent with mortality benefit in the trial being restricted to patients with missed TB diagnoses (and therefore not included in this analysis) [11]. Median time to death was 12 days (IQR, 5–27 days), and 71.7% (71/99) occurred during admission, with 9 (9.1%) within 48 hours and 32 (32.3%) within 7 days of admission (Supplementary Figure 1).

Mortality was lowest in patients with negative urine TB tests (19.7% by 56 days), compared to disseminated urinary TB with 1 (30.5%) or 2 (40.7%) positive urine tests (*P* = .018). Reduced functional ability (lower Karnofsky score, self-reported reductions in mobility, self-care, and usual activities) and poor nutritional status (MUAC and BMI) were strongly associated with mortality (Table 3). Mortality was also associated with lower CD4 count, hemoglobin, and renal impairment, although not with WHO danger signs.

**Table 3.** Factors Associated With Time to Death Over 56 Days

			Univariable Analysis				Multivariable Analysis			
Characteristic	Deaths (n = 99)	Mortality Risk, %	HR	Lower CI	Upper CI	*P* Value	HR	Lower CI	Upper CI	*P* Value
Age	…	…	1.02	1.00	1.03	.073	1.03	1.01	1.05	.003
Sex										
Female	35	23.8	1	…	…		1	…	…	
Male	64	36.8	1.74	1.15	2.63	.007	1.77	1.01	2.70	.008
Site										
Malawi	54	34.8	1	…	…		…	…	…	
South Africa	44	25.5	0.78	.53	1.16	.217		…	…	
ART										
Currently taking	80	34.3	1	…	…		^a^	…	…	
Not currently taking	19	21.6	0.60	.36	.99	.035		…	…	
Duration of illness							^a^			
<7 d	28	22.6	1	…	…			…	…	
>7 d	71	36.2	1.77	1.14	2.74	.008		…	…	
EQ5D mobility^b^							…			
No problems	9	11.0	1	…	…	< .001		…	…	
Some problems	51	31.5	3.21	1.58	6.52			…	…	
Confined to bed	99	50.7	6.26	3.03	12.93			…	…	
EQ5D self-care^b^							…			
No problems	10	9.4	1	…	…	< .001		…	…	
Some problems	47	36.4	4.63	2.34	9.17			…	…	
Unable to wash/dress	42	48.8	6.86	3.44	13.68			…	…	
EQ5D usual activities^b^							…			
No problems	5	7.6	1	…	…			…	…	
Some problems	29	32.2	5.05	1.96	13.06	< .001		…	…	
Unable to perform	65	39.4	6.30	2.54	15.65			…	…	
EQ5D health score^b^	…	…	0.97	.96	.99	< .001	…	…	…	
BMI, kg/m^2^	…	…	0.96	.91	1.01	.086	…	…	…	
MUAC, cm	…	…	0.87	.82	.92	< .001	0.89	.83	.94	< .001
Karnofsky score^b^	…	…	0.96	.95	.98	< .001	…	…	…	
WHO danger sign							…			
No	51	27.9	1	…	…			…	…	
Yes	48	34.8	1.34	.90	1.99	.146		…	…	
Hemoglobin^c^, g/dL	…	…	0.86	.80	.93	< .001	0.88	.81	.95	.002
CD4 count, cells/µL	…	…	0.93	.88	.99	.022	^a^	…	…	
eGFR^c^										
≥60 mL/min	17	25.0	1	…	…		…	…	…	
<60 mL/min	15	51.7	2.45	1.23	4.93	.013				
CRP^c^, mg/L	…	…	1.01	1.00	1.01	.043	…	…	…	
WHO clinical stage							…			
1 or 2	7	20.0	1	…	…			…	…	
3	28	25.5	1.36	.60	3.12	.031		…	…	
4	64	36.4	2.14	.98	4.66			…	…	
Able to produce sputum										
No	32	40.0	1	…	…			…	…	
Yes	67	27.8	0.66	.43	1.0	.061	^a^	…	…	
TB-LAM										
Negative	29	25.9	1	…	…		…	…	…	
Positive	70	33.5	1.72	1.00	2.95	.044	…	…	…	
Urine Xpert										
Negative	49	25.3	…	…	…		…	…	…	
Positive	50	39.4	1.91	1.28	2.87	.002	…	…	…	
Urine TB score	…	…	1.84	1.05	3.24	.024	1.41	1.03	1.91	.025

Hazard ratios calculated using Cox proportional hazards models. Continuous variables were all modeled as linear after checking for departures from linearity. For continuous variables, HR is for every unit increase in the variable, except for CD4 count where HR is for every 50 cells/µL increase. Urine TB score was modeled as a continuous linear variable. All *P* values calculated using likelihood ratio testing.

Abbreviations: ART, antiretroviral therapy; BMI, body mass index; CI confidence interval; CRP, C-reactive protein; eGFR, estimated glomerular filtration rate; EQ5D, EurQol 5 dimension; HR, hazard ratio; MUAC, mid-upper arm circumference; TB, tuberculosis; TB-LAM, Determine TB-LAM Ag assay; WHO World Health Organization.

^a^Variables eliminated from the multivariable model.

^b^Variable excluded from multivariable model as distal on causal pathway to death. CRP and eGFR were excluded from the multivariable model due to >25% missing data. The following variables were excluded due to colinearity: BMI was colinear with weight; WHO stage was colinear with CD4 count; TB-LAM and urine Xpert were colinear with urine TB score. All other variables with *P* > .1 in univariable analysis were entered into the multivariable model using backwards stepwise elimination (variables exited the model if *P* > .1), n = 321.

^c^Missing data: 1 missing hemoglobin, 93 missing CRP, 225 missing eGFR.

In the multivariable model (n = 320; Table 3), mortality was independently associated with advancing age, male sex, lower MUAC, lower hemoglobin and higher urine TB score. CD4 cell count was not independently associated with mortality.

Not taking ART at admission was associated with a lower mortality than taking ART at admission in an unadjusted model (hazard ratio [HR], 0.6; *P* = .035; Supplementary Figure 2), despite those taking ART having a lower median CD4 count (39 cells/µL [IQR, 13–122 cells/µL] compared to 84 cells/µL [IQR, 29–244 cells/µL]). Although unadjusted mortality was 9.3% lower in patients from the South African site, in an adjusted model mortality was similar to those patients from Malawi (adjusted HR, 0.9; *P* = .729; Supplementary Table).

### Clinical Phenotype

**Figure 1.**
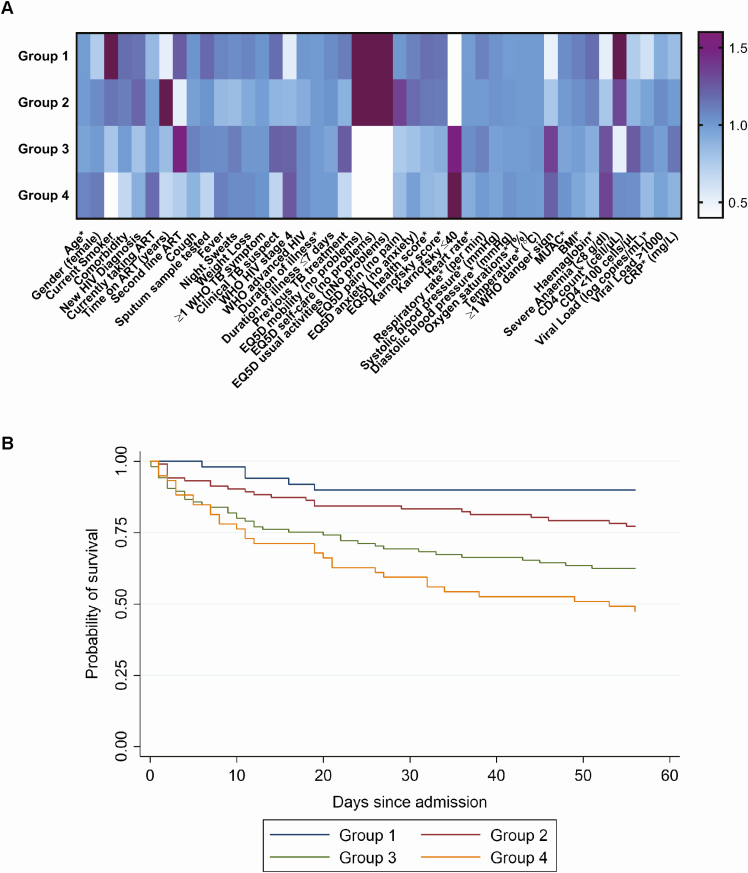
Cluster analysis clinical phenotypes of patients with human immunodeficiency virus (HIV)/tuberculosis patients and their mortality risk. *A*, Heat map comparing characteristics of clinical phenotype groups (from cluster analysis) to the overall population. Overall, n = 317; group 1, n = 51; group 2, n = 102; group 3, n = 105; group 4, n = 59. For continuous variables (marked with *), colors represent a ratio of mean or median values for the group compared to the overall mean or median. For categorical variables, the ratio is the group proportion compared to the overall proportion. Dark purple represents a ratio >1.6, and white represents a ratio <0.4. Missing data: 1 missing hemoglobin, 2 missing second-line antiretroviral therapy (ART) regimen, 93 missing C-reactive protein, 222 missing HIV viral load. EurQol 5 dimension variables are the proportion reporting “no problem.” Time on ART and second-line ART are restricted to patients reporting current ART use. *B*, Kaplan-Meier plot of time to death by clinical phenotype group. Hazard ratio compared to group 1 is 2.4 (95% confidence interval [CI], .9–6.3) for group 2; 4.5 (95% CI, 1.8–11.4) for group 3; and 6.7 (95% CI, 2.6–17.3) for group 4 (*P* < .001). Abbreviations: ART, antiretroviral therapy; BMI, body mass index; CRP, C-reactive protein; EQ5D, EurQol 5 dimension; HIV, human immunodeficiency virus; MUAC, mid-upper arm circumference; TB, tuberculosis; WHO, World Health Organization.

Cluster analysis identified 4 distinct groups of patients based on correlation of clinical features at admission. Although not informed by outcome data, mortality differed substantially between these groups (Figure 1; HRs: 2.4 for group 2, 4.5 for group 3, and 6.7 for group 4 compared to group 1; *P* < .001). Group 1 (lowest mortality risk, 9.8% [5/51]) were more likely to be ART naive, have better functional status and nutrition (higher MUAC and BMI), less severe anemia, and higher CD4 cell count. Patients in group 2 (moderate mortality risk, 22.6% [23/102]) were characterized by a longer time on ART and almost half reporting normal physical function.

Groups 3 and 4 (highest mortality risks, 37.1% [39/105] and 52.5% [31/59] respectively) patients all reported problems with usual activities, most had WHO danger signs and severe anemia, and men predominated. Median CD4 counts were 78 and 38 cells/µL, respectively. Clinical phenotype was also strongly associated with disseminated TB: groups 3 and 4 had the highest proportion of urine-positive patients (83% and 86%, respectively, compared to 55% in group 1; *P* < .001).

### Non-TB Management

Broad-spectrum antibiotics (ceftriaxone, amoxicillin-clavulanic acid, or co-trimoxazole) were given to 86.0% (277/322) of patients, with median duration 6 days (IQR, 4–7 days). 45.1% (125/277) received 2 or more different antibiotics. Ten of 94 (10.6%) patients tested were cryptococcal antigen positive. Median duration of hospital stay was 10 days (IQR, 5–15 days). Of the ART-naive patients surviving to discharge, 65.3% (49/75) commenced ART during the study, with median time to starting ART being 19 days (IQR, 9–29 days). ART was started in 66.7% (14/21) of those who had discontinued ART, and 8.3% (1/12) ART-naive patients who died during admission.

Thirty-seven of 250 (14.8%) patients discharged were readmitted to hospital during the 56-day follow-up, with readmission associated with higher mortality (24% vs 9%; *P* = .006). Outpatient attendance was also common, with patients discharged having a median of 2 (IQR, 1–4) clinic attendances by 56 days. Patients dying after discharge were less likely to attend outpatient clinics (median, 0 attendances [IQR, 0–1] compared to 3 [IQR, 1–4] for survivors; *P* < .001).

## Discussion

The main findings were that 2-month mortality in patients with HIV-associated TB was substantial (31%) despite good ART coverage, TB screening, and prompt TB treatment. Urine diagnostic tests (likely defining disseminated TB) were positive in 78.0% of TB patients, and were associated with higher mortality. Despite most patients being established on ART, advanced immunosuppression and poor virologic control were common, and we report higher mortality in patients taking ART at TB diagnosis. Hierarchical cluster analysis defined 4 distinct clinical phenotypes with highly variable mortality (9.8%–52.5%), suggesting that baseline risk profiles could be used to prioritize patients for intensified care.

Our observed early mortality is close to that estimated for HIV-associated TB from other studies [3, 15], and was high in both South Africa (25.5%) and Malawi (34.8%). This 9.3% unadjusted risk difference indicates the magnitude of effect that could be attributed to the better resources in South Africa (upper-middle income) compared to Malawi (low income). Irrespective of cause of admission, HIV-positive inpatients do badly, with early mortality risks of 20%–30% reported consistently for sub-Saharan Africa [16]. However, patients with disseminated TB do consistently worse than others. In the STAMP trial, for instance, HIV-positive inpatients without confirmed TB had 2-month mortality of 16.5%, below that of urine-positive TB, although similar to TB diagnosed only through sputum [11].

In this context, TB-LAM or urine Xpert positivity was associated with a severely ill clinical phenotype. This was also observed in a South African cohort, where markers of TB dissemination were associated with increased immune activation, which may contribute to mortality through tissue damage and organ dysfunction [15]. If we assume that positive urine results provide more rapid, less costly, and more sensitive equivalent of mycobacteremia [15, 17, 18], then these simple prognostic markers could be used to target additional interventions to reduce mortality, as well as being used for TB diagnosis.

TB patients taking ART ≥6 months had median CD4 count of only 96 cells/µL, with half having high viral loads (median, 557 000 copies/mL). This strong relationship between disseminated TB and immunosuppression in patients taking ART prior to admission suggests that urine-positive TB could be an indicator of ART treatment failure. With successful scale-up of HIV diagnosis and ART, the case mix of inpatients has changed from predominantly ART naive [19] to that reported here: 80% with a known HIV diagnosis taking ART. Interesting, this corresponds with a reversal in the prognostic value of ART prior to TB diagnosis, from beneficial [20, 21] to a risk factor for mortality [22]. Therefore, patients with TB should be immediately evaluated and managed for virological failure. However, facilities for rapid viral load testing are not universally available in inpatient settings [23]. Also, the impending roll-out of dolutegravir in the region may impact the degree of ART failure seen amongst inpatients [24].

Using both regression and cluster analysis, we found more severe functional impairment, worse nutritional status, and severe anemia being associated with both higher mortality and disseminated HIV/TB. Our findings support those reported in seriously ill HIV-positive inpatients with cough, where functional impairment (but not all WHO danger signs) were associated with poor outcomes [22]. The remarkably high mortality (50%) in those reporting severe functional impairment highlights the importance of upstream interventions to prevent TB, support early diagnosis, and improve recognition of critically ill patients in community settings. Most patients reached their critically ill state despite attending HIV-care services: Empowering patients to promptly seek care could contribute to better outcomes.

Intervening to avert mortality in HIV/TB patients after admission may be challenging (Table 4). However, only one-third of deaths occurred within 1 week, suggesting a potential window of opportunity. The best strategy for supportive care in African hospitals with high HIV and TB prevalence remains unclear, with early fluid resuscitation leading to increased mortality in patients with sepsis [25]. Although provision of broad-spectrum antibiotics in our cohort was almost universal, resistance to first-line antibiotics in study hospitals is now commonplace, especially in Enterobacteriaceae through extended spectrum β-lactamases [26]. Although Schutz et al [15] found TB, rather than bacterial coinfection, as the major cause of mortality in HIV-positive inpatients, empirical antimicrobial agents have been associated with lower mortality in advanced HIV [27]. Future strategies should account for antimicrobial resistance, and also target TB-related mortality, for instance trialing high-dose rifampicin and/or host-directed therapies [28, 29].

**Table 4.** Implications and Potential Interventions to Reduce Human Immunodeficiency Virus/Tuberculosis Mortality

Issue	Evidence for Association With HIV/TB Mortality	Possible Interventions	Further Research/Unanswered Questions
ART failure	• Higher mortality in ART-experienced patients • Low median CD4 counts and high viral loads in patients taking ART >6 mo	• Rapid screening for virological failure at admission (eg using point-of-care HIV viral load assay) • Adherence interventions or switch to second-line ART • Integrase inhibitors (few drug–drug interactions with TB medication)	• Prevalence of HIV drug resistance • Timing of ART switch in HIV/TB patients failing ART • Optimal regimen for switching
TB during early ART	• One-fifth of HIV/TB patients were within 3 mo of ART initiation • High mortality	• Improved TB screening at ART initiation • Better implementation of TB preventive therapy	• Best approach for TB screening in ART-naive patients (eg TB-LAM, Xpert, and/or chest radiography) • Implementation research for TB preventive therapy
Supportive care and comorbidities	• WHO danger signs and sepsis are common • 10% cryptococcal antigenemia • Life-threatening anemia has high mortality • 28% of deaths after discharge	• Screening, treatment, and/or prophylaxis for coinfection • Improved supportive care • More intensive follow-up postdischarge from hospital	• Prevalence of bacterial coinfection and AMR • Evidence for safety and efficacy of supportive care (eg, IV fluids and/or blood transfusion) • Impact of enhanced follow-up on outcomes, and frequency of visits
Identification of high-risk patients	• Clinical phenotype associated with high mortality • Urine diagnostics associated with higher mortality	• Predictive tools to identify patients at higher risk of mortality who may benefit from interventions (eg, clinical risk score)	• Derivation and validation of prognostic score • Use of score(s) for implementation of interventions aimed at mortality

Abbreviations: AMR, antimicrobial resistance; ART, antiretroviral therapy; HIV, human immunodeficiency virus; IV, intravenous; TB, tuberculosis; TB-LAM, Determine TB-LAM Ag assay; WHO, World Health Organization.

Cryptococcal meningitis is also a common cause of death in advanced HIV, and may be associated with HIV/TB [30, 31]. Despite exclusion of patients with altered conscious level, cryptococcal antigen positivity was >10%, supporting screening and treatment of cryptococcal infection in this population. Anemia has previously been associated with poor outcomes in HIV/TB [32], and this was also shown in this study. There are currently no normative guidelines on managing anemia or blood transfusion in HIV/TB, and further research is needed before recommendations can be made [33].

Almost one-third of deaths occurred after discharge, and those who died were less likely to attend outpatient clinics for follow-up. One-third of ART-naive patients discharged alive had not commenced ART by 2 months, despite guidelines advocating early ART. This supports more intensive follow-up and support at discharge as a possible intervention to reduce mortality. Weekly follow-up was part of an intervention that reduced mortality in patients with low CD4 counts starting ART, and warrants evaluation in HIV/TB [34].

The strengths of this study include its being nested within a TB screening trial, that management was undertaken by routine health services, and that these data reflect the high ART coverage for the Southern Africa region. Limitations include suboptimal sensitivity of the TB screening algorithms, notably for the sputum-only arm of the parent trial. Our definition of confirmed TB included some patients who were only TB-LAM positive, and we cannot exclude a small number of false positives [35, 36]. Sputum induction, which has been shown to increase the yield of sputum Xpert, was not available in this study [37]. Finally, we do not have postmortem data.

In summary, we have shown high mortality in hospitalized patients with HIV-associated TB despite current public health interventions. Urine diagnostics provide useful prognostic and well as diagnostic information, and could be used to guide the development and targeting of intensified inpatient management strategies. HIV/TB in patients in this region now predominantly affects patients established on ART with advanced immunosuppression likely to indicate ART failure. This population will be important to meet both the EndTB and Joint United Nations Programme on HIV/AIDS 2020 and 2025 targets for reducing TB deaths. Early diagnosis and management of ART failure is one of several potential interventions that could improve survival of patients hospitalized with TB. Implementation is needed in parallel with further research and upstream public health interventions.

## Supplementary Data

Supplementary materials are available at *Clinical Infectious Diseases* online. Consisting of data provided by the authors to benefit the reader, the posted materials are not copyedited and are the sole responsibility of the authors, so questions or comments should be addressed to the corresponding author.

ciz1133_suppl_Supplementary_Methods

## Notes

***Financial support.*** This Screening for TB to Reduce AIDS Related Mortality in Hospitalised Patients (STAMP) trial was funded by the Joint Global Health Trials Scheme of the UK Department of Health and Social Care, the Department for International Development, the Global Challenges Research Fund, the Medical Research Council and Wellcome Trust (number MR/M007375/1). A. G.-W. has received a Royal College of Physicians London James Maxwell Grant Prophit Fellowship, and E. L. C. has received a Wellcome Trust Fellowship (number WT200901/Z/16/Z).

***Potential conflicts of interest.*** J. J. v. O. reports grants from the London School of Tropical Medicine and Hygiene. All other authors report no potential conflicts of interest. All authors have submitted the ICMJE Form for Disclosure of Potential Conflicts of Interest. Conflicts that the editors consider relevant to the content of the manuscript have been disclosed.
