# Supplementary material for: Tuberculosis in Hospitalized Patients With Human Immunodeficiency Virus: Clinical Characteristics, Mortality, and Implications From the Rapid Urine-based Screening for Tuberculosis to Reduce AIDS Related Mortality in Hospitalized Patients in Africa
Source: Clin Infect Dis. 2019 Nov 29;71(10):2618–26. doi: 10.1093/cid/ciz1133 (PMC7744971; doi:10.1093/cid/ciz1133)
Supplement: ciz1133_suppl_Supplementary_Methods [file ciz1133_suppl_supplementary_methods.docx]

**Supplementary methods**

Study procedures

The study was undertaken at Zomba Central Hospital, Southern Malawi, and Edendale Hospital, KwaZulu-Natal, South Africa). Data were collected on clinical presentation (including TB symptoms), past medical history including HIV status and previous TB, physical examination (including vital signs, WHO HIV staging, body mass index (BMI), mid-upper arm circumference (MUAC) and Karnofsky functional score) and the EuroQol 5 Dimension (EQ5D) quality of life questionnaire. ART status was verified by checking prescriptions or health records. Clinical events during admission were recorded, and blood was also collected for CD4 cell count, haemoglobin and plasma storage.

TB screening test results were reported to the attending clinical team (reported as positive, negative or not done to maintain masking to trial arm) who made all decisions regarding TB treatment and other management. Clinicians could request further investigations (for example Xpert testing of sputum, chest radiography or cerebrospinal fluid examination) or prescribe empirical TB treatment. The study team were not involved in the management of patients, which was done as per local practice and guidelines. First-line TB treatment was the standard four-drug regimen and first line antiretroviral therapy (ART) was efavirenz-based using a fixed-dose combination pill at both sites. Patients were followed-up at 56-days post enrolment through in person interview. Patients who did not attend their follow-up appointment contacted by telephone and/or home visit, with interview of next of kin to establish vital status if necessary.

All TB assays were performed as per manufacturer’s instructions. A grade-one cut-off defined positive TB-LAM tests (using the manufacturers post-2014 reference card) and all TB-LAM results were read by a second, blinded, reader for quality assurance. Stored urine was tested with TB-LAM by thawing an aliquot of unconcentrated urine at room temperature, and with Xpert by thawing an aliquot of frozen concentrated urine (produced by concentrating approximately 40-50mls of urine by centrifugation). Stored urine was tested using the same procedures for testing urine in real-time, and followed manufacturer’s instructions.

HIV viral load testing was done retrospectively from frozen plasma using the Xpert quantitative HIV viral load assay on a consecutive sub-sample of HIV/TB as part of another sub-study. Cryptococcal antigen testing was done using IMMY CrAg LFA (Cryptococcal Antigen Lateral Flow Assay) on a random sample of patients in the same sub-study. Serum creatinine was measured by the South African National Health Laboratory Service (NHLS).

Definitions

Anaemia was defined using the WHO criteria: no anaemia (haemoglobin [Hb] concentration ≥13.0 g/dL for males, ≥12.0 g/dL for females), mild anaemia (11.0–12.9 g/dL for males, 11.0–11.9 g/dL for females), moderate anaemia (8.0–10.9 g/dL for males and females), severe anaemia (<8.0 g/dL for males and females), with Hb <6.5g/dl classified as life threatening [1,2]. Co-morbidity was defined as self-reporting of diabetes or renal, liver or cardiovascular disease. WHO danger signs were any one of heart rate >120 beats per minute (bpm), respiratory rate >30 per minute, temperature >39 degrees Celsius or being unable to walk unaided. Sepsis was defined as systolic blood pressure <90 mmHg, oxygen saturations <90%, respiratory rate >30 per minute or GCS <15 (adapted from Sepsis-3 as some measurements were unavailable) [3]. WHO TB symptom screen is one of current cough, fever, night sweats or weight loss. Advanced HIV is defined as CD4 cell count <200 cells/µl or WHO stage 3 or 4 disease [4]. Rifampicin resistant TB was defined as the presence of *rpoB* mutations on Xpert that were confirmed on repeat Xpert testing of the same or a repeated sample [5]. Estimated glomerular filtration rate (eGFR) was calculated using the 4 variable Modification of Diet in Renal Disease (MDRD) formula in the subset of patients with serum creatinine levels measured by clinicians [6].

Statistical analysis

Patients were characterised using simple descriptive statistics. Proportions were compared using Chi-squared, Fisher’s exact and McNemar’s tests as appropriate, medians were compared using Wilcoxon rank-sum tests and means using unpaired t-tests.

Time-to-mortality was calculated using survival analysis and Kaplan-Meier curves. Time was censored at 56-days post enrolment, or at the time last seen alive for those lost to follow-up. For mortality risk, patients lost to follow-up were assumed to be alive at 56-days. Schoenfeld residual plots and were used to identify departure from the proportional hazards assumption. If variables were colinear, only one was included in the model. Variables with >25% missing data were excluded from multivariable models and no imputation of missing data was done (C-reactive protein and estimated glomerular filtration rate [eGFR]). Variables considered too proximal to death which were excluded in the multivariable models were: EQ5D mobility, self-care and usual activities and Karnofsky functional score. Likelihood ratio testing was used to compare models and assess for interactions. In the final models, there was no evidence for any significant interaction between variables.

For the unsupervised analysis of clinical phenotype, all continuous variables were reduced using principal components analysis to the smallest number of components which have an eigenvalue ≥1 and explain >50% of the variance. Hierarchical cluster analysis was then used on the reduced principal components and remaining categorical factors, using Ward’s average linkage and selecting the number of clusters based on Duda and Calinski stopping rules (stopping at the number of clusters with largest pseudo-F and pseudo-T-squared values) [7]. The identified clusters were validated using kmeans cluster analysis. To describe the phenotypes of the cluster groups, a ratio of mean or median values for the group compared to the overall mean or median was calculated for continuous variables, and for categorical variables a ratio of the group proportion compared to the overall proportion.

Supplementary Figure 1: Timing of deaths, stratified by in-patient and out-patient

Stacked bar chart showing timing of deaths in days after admission, stratified by if death occurred as inpatient (green) or as outpatient (white). Total number of deaths is 99.

Supplementary Figure 2: antiretroviral therapy (ART) status and mortality

Kaplan-Meier plot of survival by antiretroviral therapy (ART) status. Patients taking ART at admission (blue, solid) includes all patients reporting current ART use. Hazard ratio 0.6 for mortality in ART naïve compared to ART experienced patients (95% confidence interval 0.4-1.0), p=0.035.

Supplementary Table 1: Multivariable model for mortality by study site

| **Characteristic** | **Univariable** | | | |
| --- | --- | --- | --- | --- |
|  | **HR** | **Lower CI** | **Upper CI** | **p-value** |
| **Site** Malawi | 1 |  |  |  |
| South Africa | 0.92 | 0.56 | 1.50 | 0.729 |
| **Age** | 1.02 | 1.00 | 1.04 | 0.075 |
| **Sex** Female | 1 |  |  |  |
| Male | 1.98 | 1.25 | 3.11 | 0.003 |
| **ART** Currently taking | 1 |  |  |  |
| Not currently taking | 0.64 | 0.38 | 1.08 | 0.097 |
| **Body Mass Index (BMI)** | 1.01 | 0.95 | 1.07 | 0.806 |
| **Cough** |  |  |  |  |
| No | 1 |  |  |  |
| Yes | 1.46 | 0.91 | 2.34 | 0.115 |
| **WHO danger sign** |  |  |  |  |
| No | 1 |  |  |  |
| Yes | 0.67 | 0.40 | 1.14 | 0.144 |
| **Haemoglobin (g/dL)** | 0.99 | 0.98 | 1.00 | 0.009 |
| **Urine TB score** | 1.23 | 0.90 | 1.71 | 0.195 |
| **Karnofsky** | 0.96 | 0.94 | 0.98 | 0.001 |
| **CD4 count (cells/µL)** | 1.00 | 1.00 | 1.00 | 0.324 |
| **Clinical TB suspect** |  |  |  |  |
| No | 1 |  |  |  |
| Yes | 0.83 | 0.49 | 1.38 | 0.468 |

Model includes variables that differed between study sites and other confounders for mortality. N=321. ART is antiretroviral therapy, BMI Body Mass Index, CI confidence interval, HR hazard ratio, WHO World Health Organization.

**Supplement References**

1. National Institute of Health. Division of AIDS (DAIDS) Table for Grading the Severity of Adult and Pediatric Adverse Events Version 2.1. 2017.

2. World Health Organization. Haemoglobin concentrations for the diagnosis of anaemia and assessment of severity. Geneva: 2011.

3. Singer M, Deutschman CS, Seymour CW, et al. The Third International Consensus Definitions for Sepsis and Septic Shock (Sepsis-3). JAMA **2016**; 315:801.

4. World Health Organization. Guidelines for managing advanced HIV disease and rapid initiation of antiretroviral therapy. Geneva: 2017.

5. World Health Organization. Xpert MTB/RIF Implementation Manual. Geneva: World Health Organization, 2014.

6. Levey AS, Bosch JP, Lewis JB, Greene T, Rogers N, Roth D et al. A More Accurate Method To Estimate Glomerular Filtration Rate from Serum Creatinine: A New Prediction Equation. Ann Intern Med **1999**; :461–470.

7. Everitt B. Cluster Analysis. Wiley, 2011.
